# Telescopic Denture

**DOI:** 10.2174/1874210601812010246

**Published:** 2018-03-30

**Authors:** Mohammad Ayham Hakkoum, Ghassan Wazir

**Affiliations:** Department of Removable Prosthodontics, Faculty of Dentistry, Damascus University, Damascus, Syrian Arab Republic

**Keywords:** Denture retention, Overlay denture, Overdenture, Denture design, Dental abutments, Crowns

## Abstract

**Aim:**

This article explains the concept of telescopic denture.

**Procedure:**

It describes the different types of telescopic attachment (or double crown), and provides an overview of the advantages and the disadvantages of this type of prosthodontic treatment.

**Conclusion:**

The indications and the clinical applications of telescopic denture are discussed.

## 1 INTRODUCTION

Many types of retentive systems are used in removable partial dentures [1-3]. Also, several types of attachments are used with overdentures [4, 5].

One of those systems that may be used as retainers for partial dentures or as attachments for overdentures is the double crown system (or telescopic attachments). A telescope crown was patented in 1873 by Dr. J. B. Beers [6]. Telescoped bridgework was reported in The American System of Dentistry 1887 and F. A. Peeso published in 1894 his system of removable bridgework which was supported by telescope crowns [7, 8]. Telescopic crowns are widely used in some European countries such as Germany and Sweden [9], and in the East of Asia.

**Fig. (1):**
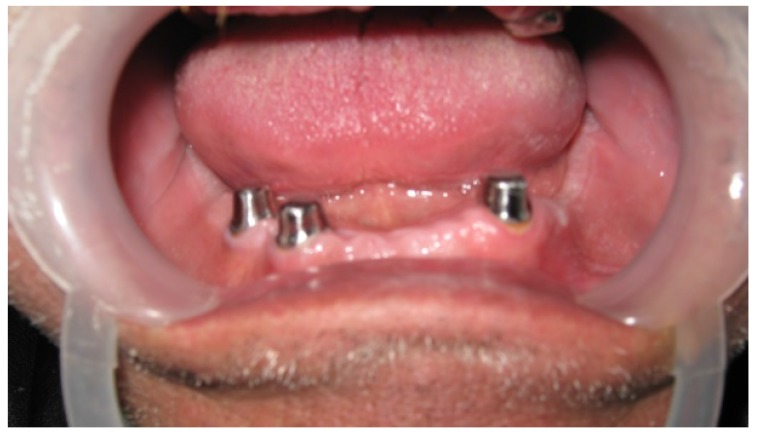
Inner telescopic crowns.

**Fig. (2):**
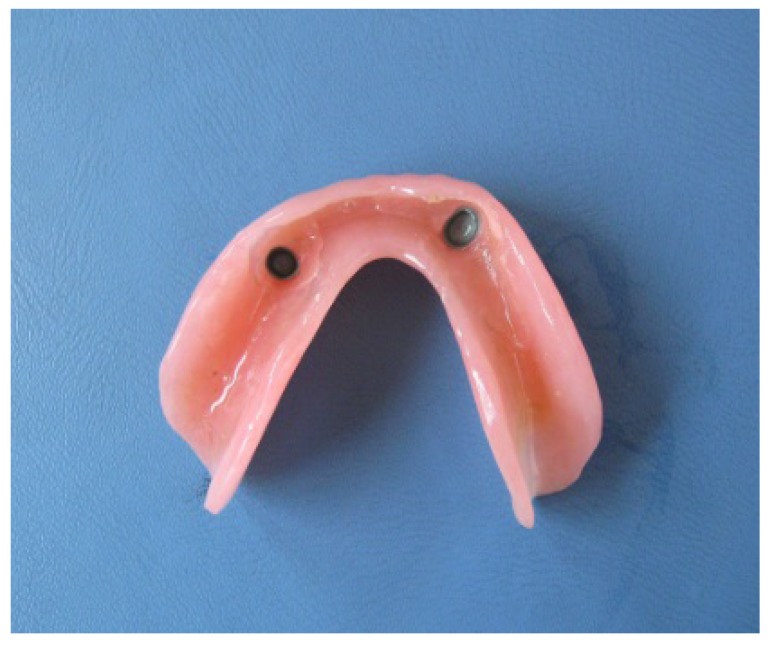
Outer telescopic crowns attached into denture base.

The term (telescopic denture) refers to the type of prosthesis that includes double crowns as retainers or attachments. These retainers (or attachments) consist of 2 crowns; primary or inner crown which is cemented to the abutment (Fig. **1**), and secondary or outer crown which is attached to the denture (Fig. **2**) [10-15]. 

The external surface of the outer crown may have the anatomic shape of the natural tooth [15], or it may be as a simple coping without anatomic landmarks [8, 16]. Retention is achieved by the fitting of the outer crown on the inner crown [14, 17-20].

The purpose of this article was to give a clear idea of telescopic denture as an available choice of prosthodontic treatment. 

## 2 TYPES OF TELESCOPIC ATTACHMENTS

Many types of telescopic attachments have been introduced to fulfill the specific requirements of clinical cases:

1- Cylindrical crowns.
2- Conical crowns.
3- Resilient designs.
4- Modified designs.

### 2.1 Cylindrical Crowns

**Fig. (3):**
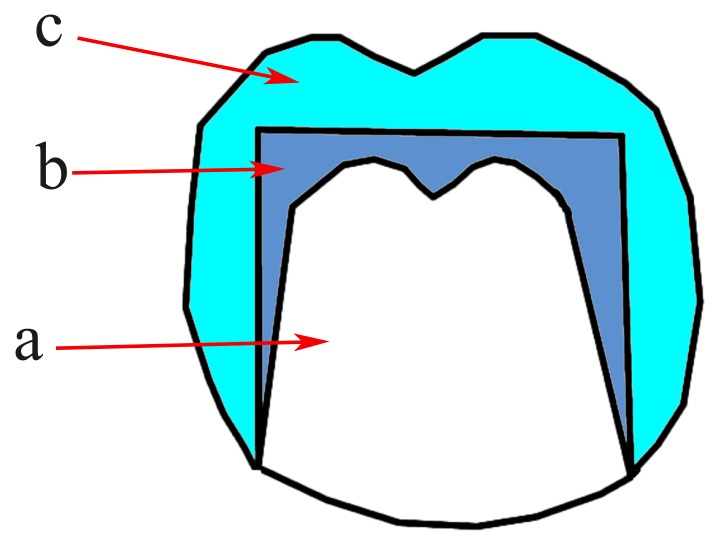
Cylindrical telescopic crown: a- Prepared abutment, b- Inner crown, c- Outer crown.

This type is the original form of telescopic crowns which is characterized by the parallel-sided inner crowns (Fig. **3**) [21]. Retention results from the friction between the inner and the outer crowns [20, 22]. This design was developed by Haupl and Böttger as mentioned by Langer and Behr [13, 23].

Cylindrical design has the advantages of great retention [24], and good esthetics in the marginal area [23]. However, the fabrication of these crowns is very difficult because the perfect and accurate fit is required between the crowns [25]. Moreover, as a result of the constant friction, the wear rate of the metal surfaces increases and a lever action occurs [20].

Therefore, this type can be used only on abutments with sound supporting tissues when there is a need for much retention.

### 2.2 Conical Crowns

This type is a modification of the previous system and was developed by K. H. Körber as mentioned by Langer, Hulten, Shiba, and Behr [9, 11, 13, 23].

**Fig. (4):**
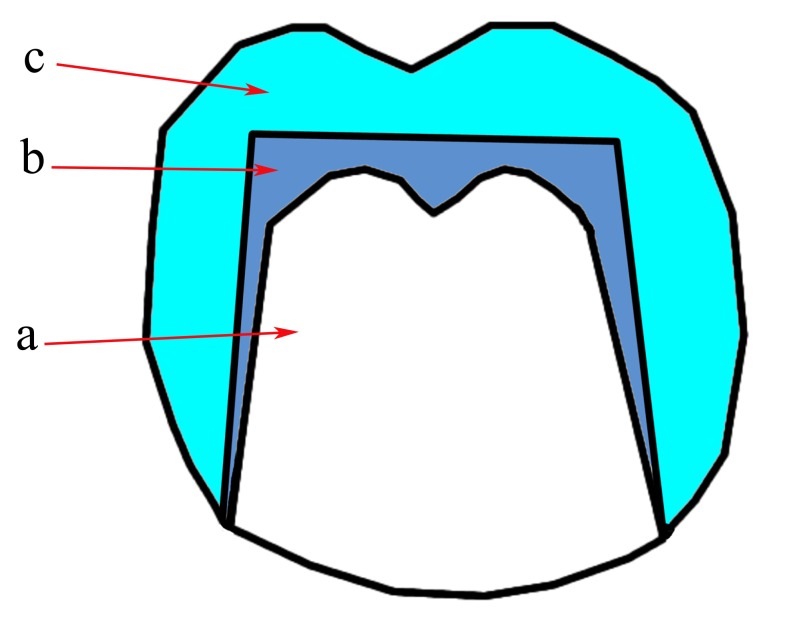
Conical telescopic crown: a- Prepared abutment, b- Inner crown, c- Outer crown.

The inner crown has a cone-like shape (Fig. **4**) [11]. So, the axial surfaces of it are tapered occlusally in a specific angle called the convergence angle (or taper) [20, 25]. Retention is obtained by the wedging action. The smaller the convergence angle the greater the retention force will be [10, 13, 22].

A convergence angle of 6^o^ is recommended. It results in a retention force of 5-10 N [23]. Shiba mentioned that 4^o^-8^o^ angle may be used according to the crown length and the physiologic movement of the abutment [11]. Some authors recommended 2^o^ angle to maintain accepted retention [20, 24, 25].

The conical type is used more widely than the cylindrical design because it is less difficult in fabrication [25], and less harmful to abutments and their supporting tissues [17, 20]. This design also has the advantage of determining the forces that would be applied to each abutment by selecting the convergence angle according to the clinical situation [12, 13]. However, the conical design has the disadvantage of retention decrease after a period of use [20, 24, 26].

### 2.3 Resilient Designs

They may be called the non-rigid designs, as they allow some freedom in the vertical and rotational movements between the inner and the outer crowns [12, 13, 27]. That may be achieved by some modifications in the inner crown, the outer crown, or both. These modifications result in reduction of the intimate contact and creation of a space between the inner and the outer crowns.

Marburg double crown is a well-known resilient design [28, 29]. It is based on the clearance fit system where only the cervical third of the inner crown is parallel to the outer crown providing a space between the crowns. This space allows a minor lateral movement between the crowns and prevents stress occurrence [22, 28, 30].

In Hofmann and Ludwig design, the cervical half of the inner crown is parallel-sided while the occlusal half is conical with a space of 0.2-0.5 mm between the crowns in the occlusal region [12, 13, 27].

Yalisove introduced a conical design where the contact between crowns was limited to the 2 occlusal thirds, while there was a space of 0.003-0.010 inches between the crowns in the cervical third allowing the rotation of the outer crown and preventing the unfavorable friction [31].

These designs provide resilient relation between the abutment and the denture, and according to their introducers and advocators they prevent the harmful effects, harmonize with tissues elasticity, result in better distribution of forces, and increase the survival rates of abutments. Resilient designs may be advantageous in the cases of a few remaining or weak abutments, and in distal extension situations. Studies showed that resilient design was successful when used in implant-supported dentures [32, 33].

### 2.4 Modified Designs

Some systems were developed by considerable modifications in the double crown concept. They mostly depend on the merging of telescopic system with another type of attachment:

- Magnotelescopic crowns [34, 35].
- O-ring coping attachment [36].
- Prefabricated telescopic attachments [13, 37, 38].

## 3 TELESCOPIC DENTURE ADVANTAGES

### 3.1 Good Retention and Stabilization

The great retentive force result from the fitting and extensive contact between the surfaces of the inner and the outer crowns [7, 8, 12, 25, 39-41].

### 3.2 Secondary Splinting Action

This is due to the accurate relation between the inner and the outer crowns, as they are rigidly connected to the denture base [7, 10, 11, 37].

### 3.3 Transferring Occlusal Forces through the Long Axes of Abutments

As the telescopic crowns surrounding abutments completely, so the occlusal forces are transmitted to abutments through their long axes [12, 39].

### 3.4 Creation of a Common Path of Insertion

That can be easily provided by the parallelism of inner crowns even if the abutments are tilted [11, 37, 42].

### 3.5 Hygienic Advantages

The telescopic attachment provides accessibility to the gingival tissues of the abutment allowing effective home care and good oral hygiene. Moreover, the good fitting of the inner crown on the abutment protects it from caries and thermal irritation [11, 13].

### 3.6 Esthetic Advantages

Using double crowns as retentive elements allows better esthetics than clasps [11, 39]. Good esthetics can be provided by using ceramic faces and a suitable color selection.

### 3.7 Patient Satisfaction

Many authors reported good patient satisfaction rates with telescopic dentures [7, 8, 10, 37, 41-44].

### 3.8 Repair and Adjustment Ability

Telescopic dentures can be easily repaired even when an abutment is lost [11, 25, 37, 45].

## 4 TELESCOPIC DENTURE DISADVANTAGES

### 4.1 Complicated Procedures

Telescopic dentures fabrication requires very complicated clinical and laboratory procedures. That results in a long treatment period and increased cost [13, 42, 46].

### 4.2 Retention Related Problems

It may be difficult to achieve the exact retention required between the 2 crowns. Moreover, the denture retention may be evaluated only after cementation of the crowns [14, 46].

Also, the retentive force between the crowns decreases after a period of use. That results from the repeated insertion and removal of the denture and the wear of crowns metal [24, 26, 47, 48].

### 4.3 Cervical Caries

The failure in providing the accurate fit of the crowns or the poor oral hygiene may lead to the occurrence of cervical caries [9, 25].

### 4.4 Esthetic Problems

Esthetic problems and difficulties may arise such as the show of crowns metal, or the overcontouring of the crowns [11, 42, 46].

### 4.5 Technical Failures

Technical failure is one of the primary problems associated with telescopic denture. Many studies reported high rates of technical failures in this type of prostheses [21, 23, 25, 43, 46, 49].

Technical failures may be loss of cementation, loss of facings, or fracture of artificial teeth, the metal framework, or the denture base [23].

### 4.6 Critical Need for Follow-up

Follow-up, periodic evaluation and maintenance are necessary to overcome the problems related to technical failures, cervical caries, and retention [21, 25, 50].

## 5 INDICATIONS OF TELESCOPIC DENTURES

1- Few remaining or unfavorably distributed abutment teeth [15, 25, 37, 44, 51].
2- When the abutment teeth need to be covered by crowns because of extensive caries or poor contour [12, 39].
3- Abutment teeth with guarded prognosis [7, 25, 46].
4- Advanced periodontitis cases [10, 52, 53].
5- When it is difficult to find a suitable path of insertion as in the case of unparallel abutment teeth [17, 37, 42].
6- Oral cancer patients [54, 55].
7- Connecting natural teeth to implants [56].
8- Occlusal reconstruction cases [41].
9- Patients with poor manual dexterity [22, 33, 54].

## 6 CLINICAL APPLICATIONS

Telescopic prostheses are widely used in clinical situations. They are not limited to a single type or design as there are several possibilities and patterns of the double crown and the telescopic denture [10].

Telescopic crowns can be used as retainers for partial dentures instead of clasps and precision attachments. In this case, the prosthesis is called (telescopic partial denture) which is usually fabricated with metal framework [12, 39].

Also, telescopic crowns are indicated as attachments for overdentures. Then the prosthesis is called (the telescopic overdenture) which is indicated generally where there are a few remaining teeth. These overdentures may be fabricated with acrylic base [7, 8].

In addition to using them with natural teeth, telescopic dentures may be supported by implants [32, 33].

Moreover, telescopic dentures may be a good choice to connect implants to natural teeth [57].

## 7 STUDIES ON TELESCOPIC DENTURE

Many studies were done to investigate telescopic dentures. A large number of these studies evaluated the retentive force associated with this type of prostheses. That is expected as telescopic crowns are basically retentive elements.

Arnold *et al*. [58] compared the retentive forces of different designs of double crowns. They found that telescopic crowns with additional retention elements had the highest retention forces. Çelik Güven *et al*. [59] analyzed the retention forces of double crowns with various fabrication methods and materials. They observed the best results when zirconia primary crown and electroformed secondary crown were used. Nakagawa *et al*. [60] investigated the effect of telescopic crowns taper on the retentive force and found that the retentive force decreased when the taper increased. Wagner *et al*. [61] evaluated the retention load of telescopic crowns. Polyetheretherketone (PEEK) crowns on cobalt-chromium primary crowns were used in their study. They concluded that telescopic crowns made of PEEK had stable retention load.

Several studies evaluated the electroplated telescopic retainers with zirconia primary crowns. Schwindling *et al*. [62] investigated the clinical outcomes of this type of telescopic denture and found favorable survival rate and good outcomes. Also, Schwindling *et al*. [63] evaluated the effect of this type of telescopic denture on Oral Health Related Quality of Life (OHRQoL). They found that OHRQoL was improved.

The effect of telescopic dentures on Oral Health Related Quality of Life (OHRQoL) was evaluated in other studies such as Elsyad and Mostafa study [64]. They found that mandibular telescopic distal extension removable partial dentures were associated with improved oral health related quality of life.

Some studies investigated the use of telescopic crown attachments in implant-supported dentures. Yunus *et al*. [65] results showed that mandibular implant-supported overdentures retained with telescopic attachments improved OHRQoL, patient satisfaction, and masticatory performance. Also, peri-implant soft tissue response and implant stability were favorable.

## 8 DISCUSSION

The telescopic denture is a unique type of prosthodontic treatment. Several designs of double crowns were developed. The selection of the design depends on the clinical situations and the dentist preference about using rigid or resilient type.

Telescopic dentures have many advantages. However, They are associated with some disadvantages. Therefore, dentists should be careful during treatment planning and the following stages. They must thoroughly evaluate the expected advantages and disadvantages keeping in mind the higher cost and the long time needed for the fabrication of telescopic denture. When planning to provide a patient with a telescopic denture the dentist should inform the patient about the long period required for treatment and should give a clear explanation about the denture that would be delivered to the patient. All clinical and laboratory steps should be accurate. Sufficient reduction of tooth structure is necessary. Otherwise, over contouring is inevitable. Dental technician plays an important role in success or failure of telescopic denture. Only skilled well equipped technician can fabricate successful telescopic dentures.

Several materials (and combinations of materials) were used for making telescopic crowns such as precious and non-precious metal alloys, zirconia, and PEEK. Further long-term studies are needed to evaluate the success of these materials and to develop new materials and designs of telescopic crowns.

## CONCLUSION

Telescopic denture should be kept in mind during treatment planning of cases requiring prosthodontic rehabilitation. It has many advantages and some disadvantages which must be evaluated carefully according to the clinical situation. The availability of various types of these dentures allows their use in a wide range of cases; especially those with a few remaining teeth or with specific complications. Further long-term research is still needed.

## CONSENT FOR PUBLICATION

Not applicable.
